# Limb-related sensory prediction errors and task-related performance errors facilitate human sensorimotor learning through separate mechanisms

**DOI:** 10.1371/journal.pbio.3002703

**Published:** 2024-07-03

**Authors:** Anushka Oza, Adarsh Kumar, Apoorva Sharma, Pratik K. Mutha

**Affiliations:** 1 Center for Cognitive and Brain Sciences, Indian Institute of Technology Gandhinagar, Gujarat, India; 2 Department of Mechanical Engineering, Indian Institute of Technology Gandhinagar, Gujarat, India; 3 Department of Biological Engineering, Indian Institute of Technology Gandhinagar, Gujarat, India; German Primate Centre Leibniz Institute for Primate Research: Deutsches Primatenzentrum GmbH - Leibniz-Institut fur Primatenforschung, GERMANY

## Abstract

The unpredictable nature of our world can introduce a variety of errors in our actions, including sensory prediction errors (SPEs) and task performance errors (TPEs). SPEs arise when our existing internal models of limb-environment properties and interactions become miscalibrated due to changes in the environment, while TPEs occur when environmental perturbations hinder achievement of task goals. The precise mechanisms employed by the sensorimotor system to learn from such limb- and task-related errors and improve future performance are not comprehensively understood. To gain insight into these mechanisms, we performed a series of learning experiments wherein the location and size of a reach target were varied, the visual feedback of the motion was perturbed in different ways, and instructions were carefully manipulated. Our findings indicate that the mechanisms employed to compensate SPEs and TPEs are dissociable. Specifically, our results fail to support theories that suggest that TPEs trigger implicit refinement of reach plans or that their occurrence automatically modulates SPE-mediated learning. Rather, TPEs drive improved action selection, that is, the selection of verbally sensitive, volitional strategies that reduce future errors. Moreover, we find that exposure to SPEs is necessary and sufficient to trigger implicit recalibration. When SPE-mediated implicit learning and TPE-driven improved action selection combine, performance gains are larger. However, when actions are always successful and strategies are not employed, refinement in behavior is smaller. Flexibly weighting strategic action selection and implicit recalibration could thus be a way of controlling how much, and how quickly, we learn from errors.

## Abstract 2

Humans learn from errors in their movements. This study shows that learning mechanisms triggered by limb-related sensory prediction errors and task-related performance errors are dissociable. While prediction errors drive implicit updates to motor plans, performance errors trigger deliberative selection of error-reducing actions.

## Introduction

Humans often have to perform actions under challenging and changing conditions. For example, a golfer may have to tee off against a constant breeze; a dancer may be required to perform while wearing a new, heavier costume; or a patient may have to adjust their gait to compensate for an emerging neurological disorder. Understanding how we adapt our actions to such changes and delineating the neural systems that support such learning has been a major goal in cognitive neuroscience. Laboratory tasks often simulate such perturbing conditions using various novel visual [1–3] or dynamic [4,5] environments that induce errors in our movements. These perturbations can create a mismatch between the intended and actual sensory consequences of action or sensory prediction errors (SPEs). SPEs are essentially limb-related execution errors that result from a miscalibrated internal model of the properties of the body and the environment. At the same time, perturbing environments can bring about a failure to accomplish the intended task goal (task performance errors, TPEs). While years of work has demonstrated that we can learn to adjust our motor plans to account for such perturbation-induced errors, a gap exists in our understanding of the relative influence of various errors and the mechanisms they stimulate to enable such learning.

Experimental investigations and theoretical models suggest that SPEs drive iterative changes in motor plans by implicitly updating our internal models of the relationship between actions and their sensory consequences [6–8]. Implicit learning driven by SPEs has some distinct features: it evolves slowly [9], asymptotes similarly for different error magnitudes [10,11], can be quite inflexible [12], and is impervious to verbal instruction [3,6]. Learning from SPEs also seems to be dependent on intact cerebellar and posterior parietal circuits [1–3]. Thus, there seems to be a reasonably strong body of knowledge about how SPEs influence motor output.

In contrast, controversy exists about how outcomes such as task success or failure influence the updating of action plans. One possibility is that performance failures, or TPEs, trigger deliberative, volitional strategies to reduce perturbation-induced errors [13]. In particular, missing a reach target may make people consciously aware of the perturbation and so they might deliberatively adjust their aim to compensate for that error. However, alternative views have emerged from work examining the influence of binary success/failure information on error-based learning [14–16]. These studies have generally shown that learning is greater when TPEs occur compared to when they do not. Such findings can be explained by including a second, TPE-driven implicit process in computational models of learning [16,17]. Thus, in this framework, TPEs independently induce implicit learning, and this process additively combines with the SPE-mediated implicit component to determine the net change in action plans. A third possibility is that learning is actually only SPE-driven, but is modulated by TPEs [16,18]. Specifically, when a movement is unsuccessful, a gain factor amplifies learning driven by SPEs (or alternatively, a positive reinforcement signal associated with a successful movement dials it down). Given these competing hypotheses—one positing deployment of volitional strategies, another postulating the stimulation of an independent implicit process, and a third advocating for modulation—a clear understanding of the mechanistic effects of task outcomes has remained elusive.

In this work, we set out to address this gap and probe how different error sources influence the planning of future actions. In our first experiment, we manipulated target size and location (through target “jumps”) but kept visual feedback of hand motion (a cursor displayed on a screen) clamped towards the original target location. Aiming to the new location in the presence of the cursor clamp induced SPEs, but a binary goal-related TPE was either present or not depending on whether the cursor either hit (no TPE) or missed (TPE present) the jumped target. We found greater learning when the TPE occurred than when it did not, a finding that could be accounted for by both the “independent” and the “interacting (modulatory)” mechanistic frameworks. In our second experiment, we hypothesized that given the insensitivity of implicit learning to verbal instruction [3,6], learning should be evident even under conditions where TPEs (but not SPEs) are present, but subjects are explicitly instructed to ignore them. Note that the Interaction model predicts no learning here, but the Independent Error model predicts that learning would indeed occur. We found no learning, leading us to set aside the idea that task-related errors independently trigger implicit mechanisms. In our third experiment, we took a closer look at the idea that SPEs and TPEs interact, and tested whether the presence of a TPE leads to a default modulation of SPE-driven learning. We again used cursor clamps and target jumps to induce SPE-driven learning in the presence or absence of TPEs, but unlike Experiment 1, instructed subjects to reach to the original target location. Here, the Interaction framework predicts learning differences between conditions in which TPEs are present versus not. But, we did not find any, leading us to reject this “interaction” possibility as well. We finally turned to the possibility that TPEs set in motion deliberative strategies, and indeed found this to be the case in our fourth experiment in which target-jump-induced TPEs were present but SPEs were not.

In sum, our results fail to support the view that task-related performance failures, or TPEs, independently induce implicit learning; we rather note that implicit learning is driven only by limb-related SPEs. We also do not find evidence favoring the view that TPEs readily modulate SPE-driven learning. Rather, our results advocate that TPEs trigger time-consuming strategic processes that are responsive to verbal instruction and suggest that sustained task success reduces or eliminates strategy use during learning. Flexibly combining SPE-based implicit processes and TPE-driven strategic action selection could then be a way for the sensorimotor system to optimize how much and how rapidly we learn from errors.

## Results

### Experiment 1

In our first experiment, 2 groups of participants performed point-to-point reaching movements in 3 blocks: baseline, learning, and washout. The hand was not directly visible during the reach, but visual feedback was provided by means of an on-screen cursor (Fig A in S1 Text). On each learning trial, the target was shifted or “jumped” to a location 10° counterclockwise to the original location. Subjects were expected to reach to this new target location. For one group (“Hit”), this jump was accompanied by an increase in target size; for the other group (“Miss”), the target size was not changed (Fig B in S1 Text). Critically, on the learning trials, motion of the feedback cursor was always clamped in the direction of the original target. This clamp ensured that the cursor always missed the new (jumped) target for the Miss group. This failure to strike the target with the cursor resulted in a TPE in the Miss participants. However, for the Hit group, the increase in target size ensured that the cursor always hit it, and thus, no TPE occurred. Importantly, however, both groups experienced an SPE early on, which occurred as subjects directed the hand to (the center of) the new target (and expected the cursor to follow), but the cursor remained clamped in the direction of the original target. In sum, the Miss group experienced both an SPE and a TPE, while the Hit group experienced only an SPE.

**Fig 1:**
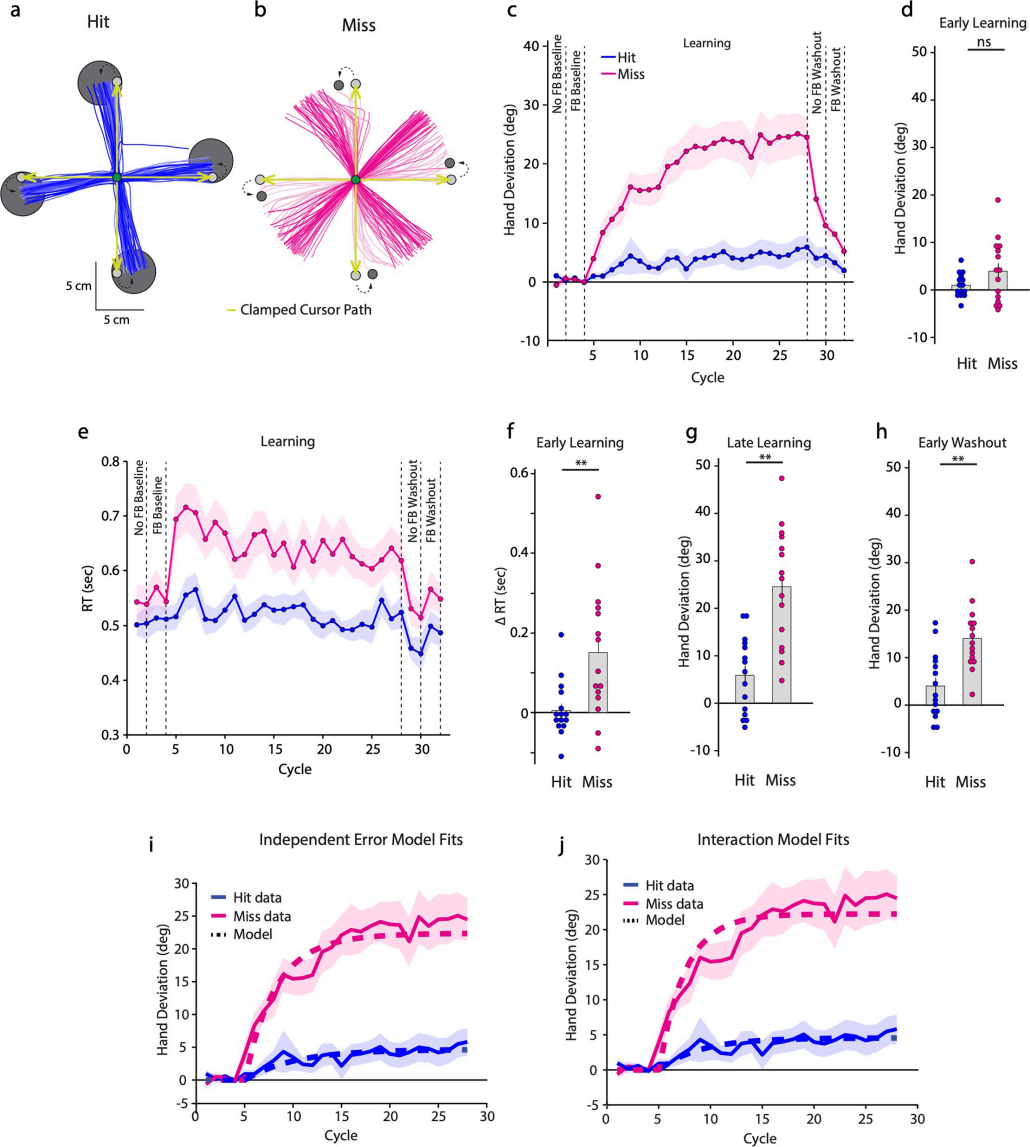
Hand trajectories of example subjects of the **(a)** Hit and **(b)** Miss groups during the learning block. Earlier trials are in lighter shades. The first 10 cm of the movement (corresponding to the target distance) are shown. The cursor path is indicated by green arrows. **(c)** Group averaged hand deviation (relative to the original target direction) across cycles of the experimental blocks. Shaded regions denote SEM. **(d)** Hand deviation during the early learning phase. **(e)** Group averaged RT across cycles of the experimental blocks. **(f)** Change in RT during the early learning phase relative to late baseline. **(g)** Hand deviation during the late learning and **(h)** early washout phases. Dots represent individual participants. **(i)** Independent Error model and **(j)** Interaction model fits to the data of Experiment 1. Solid lines represent the experimentally observed hand deviation while dashed lines represent the model fits. Shaded region denotes SEM. Source data can be found at https://doi.org/10.6084/m9.figshare.25907746.v1.

When exposed to target shifts on learning trials, Hit participants began gradually aiming towards the new target but did not go outside the target envelope (Fig 1A). In contrast, the Miss participants (Fig 1B) quickly began directing the hand to the new target, but remarkably, continued to aim well beyond it, with some saturation emerging towards the end of the learning phase. This pattern was also seen in the group data (Fig 1C). During early learning, the mean hand deviation of the Miss participants was already greater than zero (mean = 3.870 ± 1.727, CI = [0.166, 7.573]). Additionally, this deviation was greater than that seen in the Hit participants, but the group difference was not statistically reliable (Fig 1D; Hit mean: 0.910 ± 0.630°, t(17.66) = −1.61, *p* = 0.125, Cohen’s *d* = −0.588) perhaps due to higher variability in the Miss participants’ reaches.

The Miss group also showed a large change in reaction time (RT) from late baseline levels compared to the Hit group (Fig 1E). In fact, RTs continued to be elevated for the Miss participants throughout the learning block when compared to the Hit group, although a decreasing trend was seen for the Miss participants as learning progressed. Statistically, there was a larger change in RT (relative to baseline) for the Miss group during the early learning phase compared to the Hit group (Fig 1F, t(18.94) = −3.07, *p* = 0.0063, Cohen’s *d* = −1.123). Notably, RT for Hit participants did not differ from baseline at this time point (t(14) = 0.25, *p* = 0.806, Cohen’s *d* = −0.065).

As learning progressed, changes in reach direction occurred in both groups. However, by the end of learning, the Miss group had deviated much more (Fig 1G, t(23.99) = −4.878, *p* < 0.001, Cohen’s *d* = −1.781). Critically, it was not the case that the Hit group showed no change; a direct comparison between the early and late learning phases of this group revealed a clear increase in hand deviation (t(14) = −2.726, *p* = 0.016, Cohen’s *d* = −0.704). However, relative to Miss participants, this learning was conspicuously attenuated. Moreover, we found no relationship between the change in hand angle from late baseline to early learning, and the change over the remainder of the learning block (Fig A in S2 Text; Hit: R^2^ = 0.004, *p* = 0.812; Miss: R^2^ = 0.082, *p* = 0.3). The absence of such an association suggested that changes in hand angle beyond the initial stage were largely independent of changes that might have occurred early on.

During early washout (without cursor feedback), hand deviation continued to be large for the Miss group (13.978 ± 1.740°) indicating the presence of after-effects. After-effects were also evident in the Hit group (3.960 ± 1.792°, CI = [0.117, 7.803]) although they were smaller (Fig 1H; t(27.98) = −4.011, *p* < 0.001, Cohen’s d = −1.465). Importantly, these after-effects were sustained for an extended period, with both groups continuing to show large deviations even after cursor feedback was restored (Miss: 8.026 ± 0.966°, CI = [5.955, 10.097]; Hit: 3.191 ± 1.442°, CI = [0.1, 6.283]). In fact, for the Miss group, after-effects did not return to zero even after all washout trials had ended (mean = 5.114 ± 1.036°, CI = [2.891, 7.336]).

### Mathematical modeling

What drives the larger change in hand angle in the Miss **(**the group that experienced TPEs**)** compared to the Hit group (the group that does not experience TPEs)? To address this, we considered 2 mathematical models that include the influence of both SPE and TPE on iterative changes to motor output [16].

In the first **“**Independent Error**”** model, the presence of TPEs sets off an independent implicit learning process that combines with SPE-mediated implicit learning to produce the net change in motor output on a trial-by-trial basis. The equations governing the trial-wise updates in this framework are the following:

$$X_{SPE}(n+1)=A_{SPE}*X(n)+B_{SPE}*SPE(n)$$ (1)

$$X_{TPE}(n+1)=A_{TPE}(n)+B_{TPE}*TPE(n)$$ (2)

$$X(n)=X_{SPE}(n)+X_{TPE}(n),$$ (3)

where X_SPE_(n) and X_TPE_(n) denote the state of SPE-driven and TPE-driven implicit learning components on the n^th^ trial, respectively, while X(n) denotes the net motor output. A_SPE_ and A_TPE_ are retention factors that determine how much of the prior learning is carried over to the next trial, while B_SPE_ and B_TPE_ are learning rates for the SPE- and TPE-driven implicit mechanisms, respectively.

In the second “Interaction” model, TPEs cannot by themselves induce implicit learning, but can only modulate implicit learning induced by SPEs. The equations governing the trial-by-trial updates to motor output then are the following:

$$X(n+1)=G_{A}*A_{SPE}*X(n)+G_{B}*B_{SPE}*SPE(n).$$ (4)

Here, the state update is driven only by SPEs, but the modulation factors G_A_ and G_B_ (for A_SPE_ and B_SPE_, respectively) modulate this learning when TPEs are present. For fitting, the values of G_A_ and G_B_ are assumed to be one when TPEs are present, while they are allowed to vary when TPEs are absent.

#### Independent error model fit

We first fit the Independent Error model to the data from Experiment 1 (Fig 1I). To begin, since the Hit participants did not experience TPEs, only the SPE-based component (equation 1) was fit to data from the Hit participants. We obtained a good fit (R^2^ = 0.81, RMSE = 0.7553) that yielded parameter estimates as A_SPE_ = 0.8184 and B_SPE_*SPE = 0.8441. We then used these estimates in the combined model that was fit to the data of the Miss group and estimated the value of A_TPE_ and B_TPE_*TPE. Again, we obtained a good fit (R^2^ = 0.90, RMSE = 2.6833) with the parameter values as A_TPE_ = 0.7987, B_TPE_*TPE = 3.9622.

#### Interaction model fit

The Interaction model assumes that the learning is only SPE driven but the presence of a TPE brings about its modulation. To estimate model parameters, we fit this model to the data of the Miss group, while keeping G_A_ and G_B_ fixed at 1 (Fig 1J). We again obtained a good fit (R^2^ = 0.89, RMSE = 2.819), and parameter values were estimated as A_SPE_ = 0.6735, and B_SPE_*SPE = 7.2607. We then used these estimated parameter values to fit the model to the mean data of the Hit group and probe how G_A_ and G_B_ would change. We obtained R^2^ = 0.7938 and RMSE = 0.7724, while G_A_ and G_B_ were estimated to be 1.1534 (CI = [0.2147, 1.4347]) and 0.1401 (CI = [0.0249, 0.4913]), respectively (confidence intervals derived from fits to 10,000 bootstrap samples of data from this group). Importantly, the confidence intervals of the parameter values indicated that while G_A_ was not different from 1, G_B_ differed significantly from this value. This indicated that modulation of the SPE-driven learning (the B_SPE_*SPE parameter) could occur via TPEs.

Thus, our modeling effort revealed that both, the Independent Error model as well as the Interaction model could account for the differences between the Hit and Miss groups of Experiment 1.

### Experiment 2

The success of both models in accounting for the results of Experiment 1 presented a conundrum. In particular, the success of the Independent Error model suggested that it was at least mathematically possible to account for the higher learning in the Miss group via an implicit mechanism triggered by TPEs that acted in concert with a different, SPE-driven implicit process. A pivotal empirical prediction of this framework is then the following: since implicit learning is impervious to verbal instruction [3,6], some learning (change in hand angle) should occur even in conditions where only a TPE is present, but participants are explicitly instructed to ignore it. We tested this hypothesis in our second experiment. We recruited 2 groups of participants that underwent training like the Miss group of Experiment 1 (TPEs present) but were told to ignore the (10° or 20°) jump-induced TPE and reach to the original target location. Notably, as in Experiment 1, the visual cursor was clamped in the direction of the original target. Since subjects were told to move towards the original target location and the cursor also followed in the same direction, the classically defined SPE was not induced. That is, since there was no mismatch between the expected and actual cursor motion, the SPE was zero. To reiterate, in this second experiment, subjects were asked to ignore the non-zero TPE resulting from the cursor missing the jumped target while they experienced no SPE. We expected that if the TPE is capable of inducing implicit learning independently, then a change in hand angle must occur despite the instruction to ignore it.

**Fig 2:**
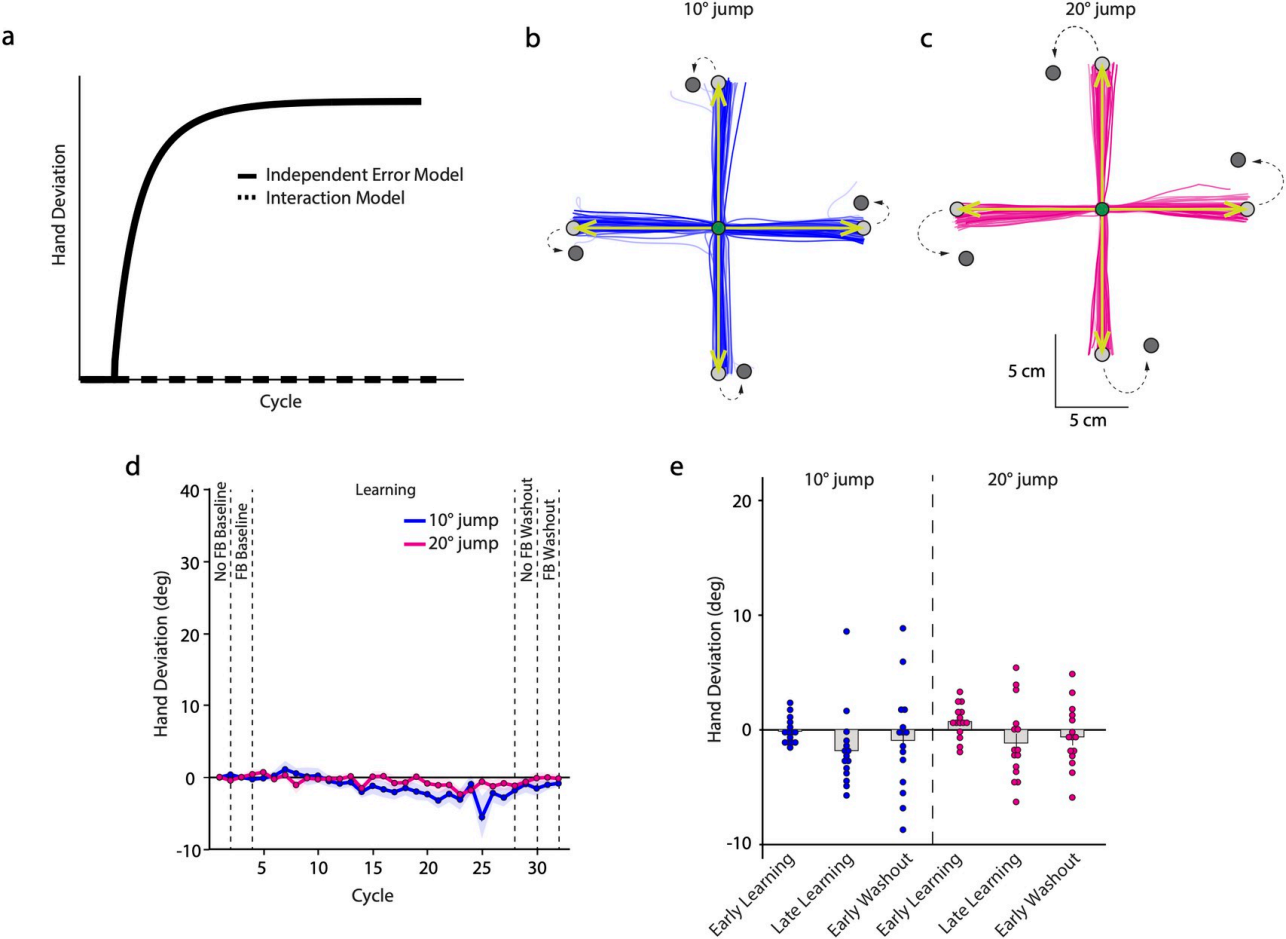
**(a)** Predictions of the Independent Error model and Interaction model for Experiment 2. Since there is no SPE, the Interaction model would predict no learning as shown by the dotted line. The Independent Error model, represented by the solid line, suggests normal learning. Hand trajectories of example subjects in the **(b)** 10° and **(c)** 20° target jump groups during the learning block. Earlier trials are in lighter shades. The first 10 cm of the movement are shown. The green arrows indicate the clamped cursor trajectory. **(d)** Group-averaged hand angle deviation across cycles of different blocks. Shaded regions denote SEM. **(e)** Mean hand deviation during the early and late learning stages, as well as the early washout phase. Dots represent individual subjects. Source data can be found at https://doi.org/10.6084/m9.figshare.25907746.v1.

What do our 2 models predict in these conditions? The Independent Error model suggests that learning would still progress because the TPE-based implicit process would not be suppressed by verbal instruction. In contrast, the Interaction model predicts no change in hand direction because in this framework, what drives learning is an SPE, which was absent in this case (Fig 2A). Remarkably, our experimental data revealed little change in reach direction. As can be seen in Fig 2B and 2C, the hand was almost always directed towards the original target, even though it had been extinguished and a new one was displayed at a location 10° (Fig 2B) or 20° (Fig 2C) clockwise from it. The absence of a change in reach direction was consistent in the group-averaged data as well (Fig 2D). During early learning, hand deviation (Fig 2E) remained close to zero for the 10° (−0.154 ± 0.29°, CI = [−0.777, 0.468]) as well as the 20° (0.687 ± 0.378°, CI = [−0.123, 1.498]) groups. This continued to be the case at the end of learning as well (Fig 2E; 10°: −1.843 ± 0.887°, CI = [−3.747, 0.06], 20°: −1.182 ± 0.863°, CI = [−3.033, 0.67]). Furthermore, after-effects on the early no-feedback washout trials were absent in both groups (Fig 2E; 10°: −0.951 ± 1.183°, CI = [−3.488, 1.585]; 20°: −0.648 ± 0.708°, CI = [−2.167, 0.871]), which continued to be the case in the second washout block with visual feedback as well (10°: −1.051 ± 0.702°, CI = [−2.556, 0.453]; 20°: −0.033 ± 0.612°, CI = [−1.345, 1.278]).

Thus, when instructed to ignore performance failures, no adaptive changes in reach behavior occurred; this would not be the case if these task errors were driving implicit tuning of reach plans. This result effectively ruled out the Independent Error framework as an explanation of the findings from Experiment 1. In other words, TPEs cannot independently induce implicit learning; rather an SPE is required for implicit learning to be set in motion.

### Experiment 3

**Fig 3:**
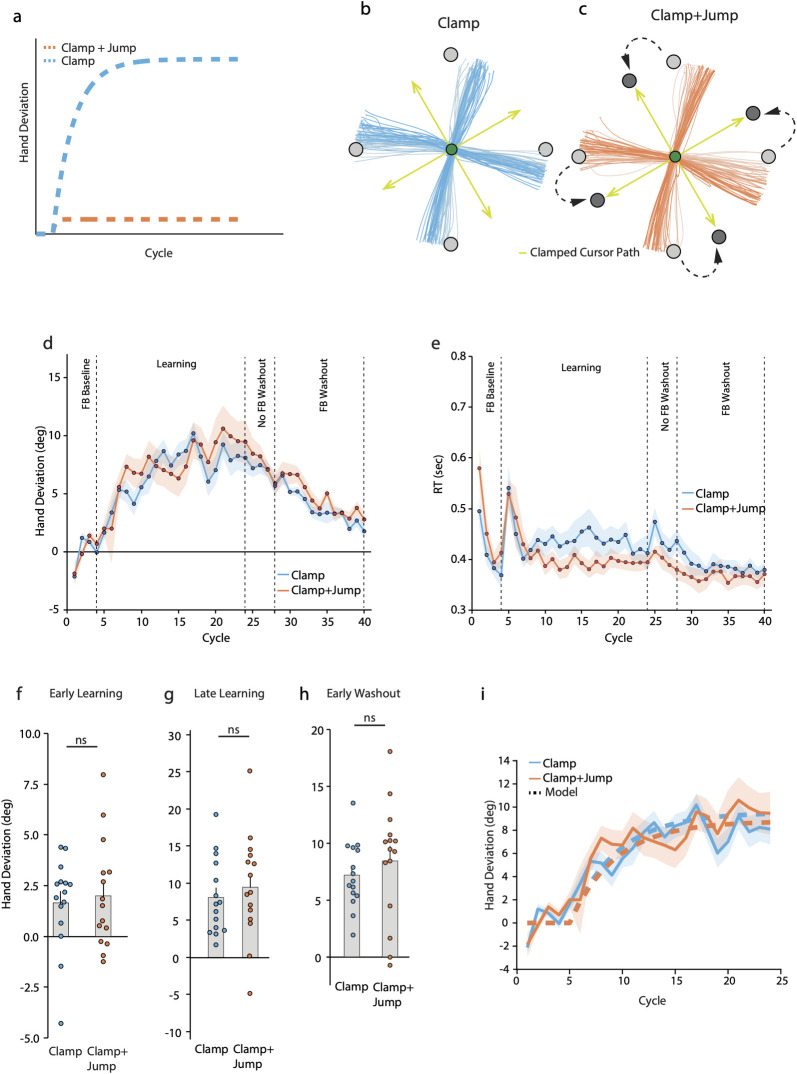
(**a**) Predictions of the Interaction model for Experiment 3. This model predicts a robust change in hand angle for the Clamp group, but attenuated learning for the Clamp+Jump group. Hand trajectories of example subjects of the (**b**) Clamp and (**c**) Clamp+Jump groups during the learning block. Earlier trials are in lighter shades. The first 10 cm of the movement are shown. The cursor path is indicated by green arrows. Group averaged (**d**) hand deviation (relative to the original target direction) (**e**) and RT across cycles of the experimental blocks. Shaded regions denote SEM. Hand deviation during the (**f**) early learning, (**g**) late learning, and (**h**) early no-feedback washout phases. Dots represent individual participants. (**i**) Interaction model fits to the data of Experiment 3. Solid lines represent the experimentally observed hand deviation (shaded region denotes SEM), while the dashed lines show the model fits. Source data can be found at https://doi.org/10.6084/m9.figshare.25907746.v1.

The results of Experiment 2 helped us disambiguate the Independent Error and the Interaction models and appeared to support the latter. However, they might not directly substantiate the Interaction model because no SPE was present in this experiment, and therefore any automatic modulation of SPE-driven learning in the presence of a TPE cannot be ascertained. To decisively test whether SPE-driven learning is influenced by TPEs, we performed a third experiment in which 2 groups of subjects reached under 30° counterclockwise error clamp conditions; we expected that this clamp-induced SPE would bring about an implicit change in hand angle in both groups. However, for one of the groups (“Clamp+Jump”), the reach target was also jumped 30° counterclockwise on each learning trial, which caused the clamped cursor to always strike it at the end of the stipulated movement distance. Since the cursor always hit the target, no TPE occurred. In the other group (“Clamp”), the target remained stationary and thus the (clamped) cursor never struck it, resulting in a TPE. This arrangement allowed us to probe if and how SPE-mediated learning induced by the error clamp changed in the presence/absence of the TPE. If the TPE modulates SPE-mediated learning as a default, there would be differences in how much the hand angle changes in the 2 groups. Such a difference is also predicted by the Interaction model. Specifically, the model predicts a large change in hand angle for the Clamp group which experiences TPEs, but attenuated learning in the Clamp+Jump group which does not experience them (Fig 3A).

Remarkably, our experimental data revealed no group differences. A clear shift in the hand angle over the learning phase was evident in both groups (Fig 3B and 3C), which was confirmed in the group data as well (Fig 3D). RT data also did not show any group differences, with RT increasing initially for both groups perhaps due to the introduction of the novel clamped feedback condition, but reducing rapidly thereafter as learning progressed (Fig 3E). A closer look at the hand deviation data revealed that the groups did not differ during the early learning phase (Fig 3F; Clamp: 1.653 ± 0.580°, Clamp+Jump: 1.999 ± 0.685°, t(27.261) = −0.385, *p* = 0.703, CI of mean difference = [−2.184, 1.492], Cohen’s d = −0.141, BF_10_ = 0.364). By the end of learning, both groups showed robust changes in hand angle (Fig 3G; Clamp: 8.087 ± 1.323°, t(14) = −4.839, *p* < 0.001, Cohen’s d = −1.249; Clamp+Jump: 9.464 ± 1.836°, t(14) = −4.081, *p* < 0.001, Cohen’s d = −1.054), but again, group differences were not significant at this time point (t(28) = −0.608, *p* = 0.548, CI of mean difference = [−6.011, 3.259], Cohen’s d = −0.222, BF_10_ = 0.396). As was the case in Experiment 1, there was no association between the change in hand angle from late baseline to early learning and the change over the rest of the learning phase (Fig B in S2 Text; Clamp: R^2^ = 0.127, *p* = 0.192; Clamp+Jump: R^2^ = 0.053, *p* = 0.409).

During the early no-feedback washout trials, large after-effects were present in both groups. Hand deviation of the Clamp group on the early after-effect trials was not different from late learning (Fig 3H; mean = 7.185 ± 0.752°, t [14] = −1.114, *p* = 0.284, Cohen’s d = −0.288), which was also the case for the Clamp+Jump group (mean = 8.434 ± 1.336°, t [14] = −1.069, *p* = 0.303, Cohen’s d = −0.276). A direct comparison of this early after-effect magnitude between the 2 groups indicated no significant differences (t(28) = −0.815, *p* = 0.422, CI of mean difference = [−1.25, 1.533], Cohen’s d = −0.298, BF_10_ = 0.443). Furthermore, after-effects persisted even when cursor feedback was restored (Clamp: 6.559 ± 0.533°, CI = [5.415, 7.703]; Clamp+Jump: 6.744 ± 1.017° CI = [4.563, 8.926]), with no significant group differences (t(21.16) = −0.161, *p* = 0.873, CI of mean difference = [−2.538, 2.167], Cohen’s d = −0.059, BF_10_ = 0.348). Remarkably, hand deviation did not reach baseline levels even on the last washout cycle for either group (Clamp: 1.762 ± 0.626°, CI = [0.420, 3.103]; Clamp+Jump: 2.779 ± 0.655°, CI = [1.373, 4.184]).

In sum, the experimental data revealed that despite experiencing a TPE, the overall performance of the Clamp group was not different from that of the Clamp+Jump group which had not encountered any TPE.

#### Interaction model fit

We then fit the Interaction model (equation 4) to the data from Experiment 3 to probe for any potential groups difference in model parameters (Fig 3I). First, we fit the model to the mean data of the Clamp group while holding G_A_ and G_B_ at 1. We obtained a good fit (R^2^ = 0.8242, RMSE = 1.3519), and model parameters were estimated as A_SPE_ = 0.9199 and B_SPE_*SPE = 0.7181. We then used these parameter values and fit the model to the data from the Clamp+Jump group to estimate G_A_ and G_B_. Note that if the estimated values of G_A_ and G_B_ from this latter fit differ significantly from 1, it would imply that the TPE experienced by the Clamp+Jump group has some influence on the SPE-mediated learning induced by the error clamp. However, we obtained G_A_ and G_B_ estimates as 0.9986 (CI = [0.9667, 1.0292]) and 1.1019 (CI = [0.6118, 1.6640]), respectively (confidence intervals derived from fits to 10,000 bootstrap samples of data from this group). This suggested that the TPE did not produce any modulatory effect.

### Experiment 4

Data from our first 3 experiments and our modeling efforts suggested that experiencing TPEs neither triggered independent implicit learning mechanisms (Experiments 1 and 2) nor did their presence automatically modulate implicit learning set in motion by SPEs (Experiment 3). Yet, as Experiment 1 showed, there was a much larger change in hand angle when TPEs were present and subjects were expected to respond to them (Miss group) compared to when they did not occur (Hit group). How then do TPEs contribute? In the Introduction, we presented 3 possibilities: first that TPEs set off independent implicit mechanisms, second that they modulate SPE-driven implicit learning, and third that they induce strategic re-aiming to rapidly reduce the imposed errors. Having ruled out the first 2, we considered the final possibility that the occurrence of TPEs triggers the use of deliberative strategies. Our final experiment was designed to test this idea. In Experiment 4, 2 groups of subjects made reaching movements under conditions in which cursor feedback was always veridical with the hand, thereby eliminating any SPE. However, one group experienced a 10° counterclockwise target jump with respect to the original target, while the other experienced a 30° clockwise jump, thus creating TPEs early on. Subjects in both groups were instructed to reach to the on-screen target, but at the end of learning and just before the after-effects block, the 30° jump group received the additional instruction that the target would stop jumping and that they should bring their hand to the original target location. This instruction was not given to the 10° jump group. We predicted that subjects in both groups would show increased RT and fleeting after-effects, classic signatures of a re-aiming strategy [19].

**Fig 4:**
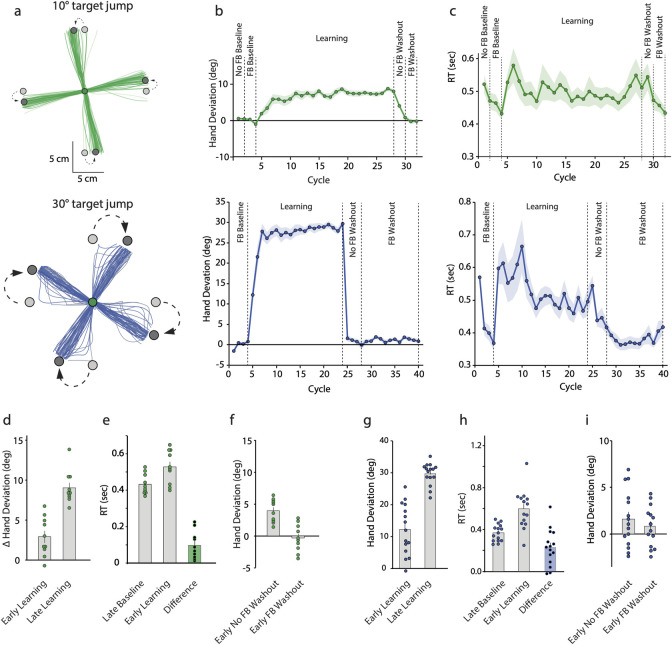
(**a)** Hand trajectories of example subjects groups during the learning block. Earlier trials are in lighter shades. The first 10 cm of the movement are shown. Group averaged (**b**) hand deviation and (**c**) RT across cycles of different experimental blocks. Shaded regions are SEM. In all panels, the upper row represents 10° target jump group while the lower row represents 30° jump group. (**d**) Change in hand angle during early and late learning relative to the late baseline stage for the 10° group, and (**g**) hand angle during early and late learning stages for the 30° group. (**e, h**) Group-averaged RT during the late baseline and early learning phases along with the RT difference. (**f, i**) Group-averaged hand deviation on the early no-feedback and feedback washout trials. In each case, dots are individual participants of the 10° (green) and 30° (blue) groups. Source data can be found at https://doi.org/10.6084/m9.figshare.25907746.v1.

Fig 4A shows the hand trajectories of example subjects from the 10° (top) and 30° (bottom) jump groups, respectively, while Fig 4B shows the group-averaged change in hand angle over the course of the experiment. It is evident that on the learning trials, the hand was directed towards the displaced target within a few trials but did not go substantially beyond it. Further, as shown in Fig 4C, RT during the learning phase increased for both groups (10°: top, 30°: bottom) and continued to remain elevated relative to late baseline trials.

Statistically, for both groups, there was a clear change in hand angle early on itself (10°: Fig 4D, t(9) = 3.76, *p* = 0.0045, Cohen’s d = 1.189; 30°: Fig 4G, t(14) = 5.352, *p* < 0.001, Cohen’s d = 1.382). The increase in RT during the early phase (10°: Fig 4E, 96.17 ± 24 ms; 30°: Fig 4H, 229.27 ± 44.55 ms) was also statistically robust (10°: t(9) = 4.015, *p* = 0.003, Cohen’s d = −1.27; 30°: t(14) = 5.146, *p* < 0.001, Cohen’s d = 1.329). By the end of learning, the mean change in hand angle relative to the late baseline stage was 9.014 ± 0.653° and 28.883 ± 1.008°, both of which were remarkably close to the magnitude of the jumps that the 2 groups experienced. Furthermore, in sharp contrast to Experiments 1 and 3, the early changes in hand angle were negatively correlated with changes over the rest of the learning block (Fig C in S2 Text; 10°: R^2^ = 0.587, *p* = 0.0097; 30°: R^2^ = 0.8, *p* < 0.001). In other words, if the initial change in hand angle was large enough to offset the error, no further learning occurred.

A dichotomy emerged in the after-effects of the 2 groups on the early no-feedback washout trials. For the 10° jump group (Fig 4F), the hand remained somewhat deviated early on (mean = 3.95 ± 0.566°, CI = [2.667, 5.227]), but for the 30° jump group (Fig 4I), there was an immediate return to baseline (mean = 1.595 ± 0.777°, CI = [−0.072, 3.262]). By the end of the first washout block however, hand deviation of even the 10° group reached near zero (mean = 0.757 ± 0.526°, CI = [−0.434, 1.948]) and the 30° group continued to maintain similar levels (mean = −0.028 ± 1.002, CI = [−2.178, 2.122]). There was no further change in hand angle over the remaining washout trials and hand deviation at the end of the feedback washout trials hovered around zero for both groups (10°: mean = −0.245 ± 0.452°, CI = [−1.266, 0.777]; 30°: 0.900 ± 0.654, CI = [−0.503, 2.303]). This suggested that after-effects of both groups were highly labile, but the 30° group showed a very rapid return to baseline since they were able to disengage from the strategy when explicitly instructed prior to the start of the washout block (also see Fig A in S3 Text). Additional analyses involving a direct comparison of this decay in these 2 TPE-only groups to that of the Hit and Miss groups of Experiment 1 confirmed the fleeting nature of the after-effects (S3 Text). This result, complemented with the robust increase in RT on the early learning trials, suggested that subjects accounted for the jump-induced TPEs via time-consuming re-aiming strategies that were rapidly disengaged when they were no longer relevant.

## Discussion

Error is believed to be the currency that drives sensorimotor adaptation to novel task conditions. We investigated the influence of different error signals on adaptive changes in motor output; we specifically asked how limb-related SPEs and task-related TPEs contribute to learning. Our findings are at odds with the notion that performance failures bring about implicit recalibration of action plans [16,17]; rather, we observe that implicit learning is engaged only by SPEs. We also do not find evidence that SPE-driven learning is obligatorily modulated by the occurrence of TPEs [18]. Rather, TPEs appear to set in motion distinct, verbally sensitive strategic action selection mechanisms to rapidly offset the error. The combination of TPE-driven strategies and SPE-driven implicit recalibration then likely determines how much and how rapidly our future action plans are tuned based on errors that we experience.

A hallmark of implicit recalibration is its imperviousness to verbal instruction [3,6]. That is, people will demonstrate implicit learning even when they are explicitly asked to ignore the error. However, rather than insensitivity, we found remarkable enslaving of TPE-induced responses to verbal directives. Consider the subjects who were expected to respond to the TPE by aiming to a new target location—the Miss group of Experiment 1 and the 2 jump groups of Experiment 4. All these participants demonstrated an increase in RT and a rapid change in hand direction on the early learning trials, both of which are signatures of deliberative strategy use to offset the error. Furthermore, during washout, the participants of Experiment 4, who experienced only jump-induced TPEs, showed transient (10° jump) or negligible (30° jump) after-effects, indicating almost immediate disengagement from the learned behavior, another robust feature of strategy use. Even more remarkable was the performance of subjects in Experiment 2, who were instructed to ignore the TPE. In complete compliance with the instruction, these subjects showed a notable lack of change in reach direction and no after-effects. Taken together, these findings refute the notion that TPEs, at least those induced via target shifts, independently induce implicit recalibration, and strongly advocate that responses to TPEs involve deliberate, strategic selection of actions that bring about a change in movement direction. This ability to either deploy or disengage strategies “on call,” despite being a time-consuming process, provides a powerful means to adjust motor behavior to task demands. While the specific nature of these strategies remains to be elucidated, potential candidates include volitional, goal-directed control [19] or mental rotation of the original movement plan [20,21].

Besides compensating for performance errors as shown here, recent work has revealed another major advantage of strategy use during learning. Notably, employing deliberative re-aiming could be the gateway to long-term storage of updates made to actions plans, and expression of the acquired memory as “savings”. More specifically, strategies that lead to rewarding task outcomes could be reinforced and recalled later, resulting in faster learning upon re-exposure to the original learning conditions [22,23]. In contrast, if strategy formation is prohibited by limiting movement planning time, by preventing exposure to performance errors, or by forcing only implicit learning, savings is blocked [24,25]. This suggests that unsuccessful task outcomes and associated strategy use are essential for forming long-term motor memories.

While our results argue that TPEs stimulate volitional strategy use, a pertinent question is why don’t TPEs induce implicit recalibration? One possibility is that for recalibration to occur, the sensorimotor system must attribute the error to some internal source, such as a limb-related execution failure [26,27]. It may be that TPEs, particularly those induced via target shifts, are instead seen as resulting from an external cause [26]. The concomitant failure to accomplish the task goal could then be seen as arising from an action selection error (rather than an execution error), which then only requires the selection of a different action on the next trial (i.e., re-aiming) rather than updates to our internal models of the body and the world. In case of consistently occurring TPEs, action selection could be enhanced by learning the task structure (extracting as much information about the environment as possible) instead of engaging the slower implicit system [19]. Once such learning has occurred, actions could be guided by representations of the outcomes they produce and what these outcomes are worth, which, in fact, is precisely what is advocated in model-based reinforcement learning theory. It is also known that such algorithms can be adjusted rapidly to account for outcome revaluation as well as changes in the evironment and action goals [19,28,29]. The quick, instruction-driven disengagement and return to near-baseline behavior on the after-effect trials of Experiment 4 is well-aligned with this idea and suggests that “learning” from TPEs could proceed in this fashion.

Is it possible that TPEs induced through target jumps also give rise to use-dependent execution biases in addition to driving deliberative changes in hand movement direction [30,31]? A careful examination of the 30° jump group of Experiment 4 may provide some insight into this because in this group, there remained a small “after-effect” of approximately 1.5° on average even after the explicit component was disengaged through instruction. The point is that subjects learn to move towards the new target location (consistently approximately 30° away from the original target in this case), and these movements could be seen as becoming “habitual.” The early set of movements to the original target at the beginning of the no-feedback washout block would then act as “probes,” and the small “after-effect” may reflect execution biases wherein reaches in the direction of the original target are slightly deviated towards the previously adapted, shifted target direction. We note however that we used 4 different target directions covering 4 quadrants on the learning trials rather than a single habitual target as in most studies of use-dependent learning. Furthermore, unlike typical experiments designed to test use-dependent biases, we did not require a large number of learning trials and no preparation time constraints were imposed. Finally, there was no consistent pattern across subjects in terms of the bias, with “after-effects” ranging from −2.396° to 6.906° (also see Fig 4I). Had this been a true bias, we would have expected a consistently positive hand angle across all subjects. The mean after-effect of approximately 1.5° was also not different from zero. Collectively, these factors do not appear to support the view that responses to TPEs induced via target jumps drive use-dependent execution biases on top of deliberative adjustments to reach direction.

Our current findings indicate that implicit learning is stimulated only when an SPE is present. In the presence of a concurrent TPE that subjects are expected to respond to, this implicit learning likely rides on top of the strategic adjustments to reach direction that the TPE sets in motion. This is precisely what we believe is the case with the Miss subjects of Experiment 1. As we noted earlier, our data from this group suggest that their early response involves explicit re-aiming to the shifted target location. This re-aiming then creates an SPE as people expect the cursor to follow the hand being aimed to the new target, while it remains clamped in the direction of the original target. The occurrence of this SPE in turn sets in motion implicit recalibration, which dominates to bring about further changes in hand angle and produce after-effects (see S2 Text for more details). Note that this SPE is just like a “classic” SPE that occurs as people move in an instructed direction while feedback about that motion is shown in another. However, our novel task design enables us to create it “on the fly” by requiring the Miss subjects to (first) respond to the TPE. Importantly, in this scheme, when strategy use is not employed—either due to verbal instruction or the absence of TPEs—net updates to action plans are determined entirely by the implicit process. This likely explains the attenuated learning in the Hit group of Experiment 1 as well as the Clamp and Clamp+Jump subjects in Experiment 3, and is perhaps also the basis for the reduced learning seen in other studies in which performance errors are eliminated [14–16].

Relatedly, in Experiment 2, although subjects experience a TPE like the Miss group of Experiment 1, they are asked to ignore it and reach the orignal target location. These subjects do not re-aim and no SPE is created since the hand and the clamped cursor are both directed towards the original target. Because no SPE occurs, no adaptive response occurs even though TPEs are present. These findings are reminiscent of classic work showing that in the presence of prediction errors, humans compulsively update their action plan in an implicit manner even if such learning bears a cost on performance [6]. In line with this result, stroke patients with lesions to right inferior frontal and dorsolateral prefrontal cortices, who demonstrate clear performance errors when exposed to a perturbation, have no problem implicitly updating their reach direction in response to SPEs [32]. Tellingly, recent work [33] suggests that the prediction error dominance may be so strong that task outcomes may have negligible influence, at least in canonical learning paradigms. Our results also strengthen this view.

We also did not find any default modulatory influence that the occurrence of TPEs produces on implicit recalibration engaged by SPEs. In Experiment 3, neither the rate nor the amount of implicit learning was influenced by the presence (Clamp group) or absence (Clamp+Jump group) of TPEs. This is at odds with recent work that also employed target jumps to induce TPEs but found evidence for modulation, albeit using within-subject, trial-by-trial designs [18,34]. How do we reconcile this difference? In typical trial-by-trial designs, TPEs are varied on each trial while the SPE is kept fixed. This results in unpredictability not just in the TPE magnitude, but also in the relationship between the SPE and TPE. This is not the case in block designs wherein these aspects remain fixed. The unpredictability in trial-by-trial designs could make the errors more salient, whereas in block designs, the errors might lose their salience. This difference might lead to modulatory effects in one but not the other. Another possibility is that for the jump-induced TPE to interact with implicit learning, the location of the shifted target needs to be in the vicinity of the original one, i.e., the TPE needs to be small. While we employed a large jump of 30° in Experiment 3, jump magnitudes were much smaller in work that showed a modulatory influence of TPEs on implicit recalibration [18,34]. In our case, the TPE may be so large that it loses its relevance and participants simply ignore it. Further, because it is always predictable, it may fail to capture attention, and implicit recalibration can proceed unhindered and to the same extent as the no-jump condition. Other experimental differences such as the timing of the jump, among others, could also be a factor. These possibilities can be disambiguated in future studies.

The lack of modulation is not to say however that the presence of binary TPEs cannot “influence” the net learning (change in hand angle). As our results clearly show, such an influence certainly arises when subjects are expected to respond to TPEs. This is specifically reflected in the fact that although both groups experienced TPEs, the hand deviation of the Miss group of Experiment 1 (which was expected to respond to the target shift and aim to the new target) was nearly 3 times larger than that of the Clamp subjects of Experiment 3 (who were instructed to ignore the jump and move their hand to the original target location). We believe that the requirement to respond to the TPE triggered strategic adjustment of aiming direction in the Miss group initially (followed by SPE-mediated implicit adjustments, as noted above) resulting in a much larger net change than the Clamp group. Barring such an instruction, the TPE carries no influence, and net learning is driven completely by the SPE, which is perhaps what transpired in the Clamp group. Our results thus point to a dichotomy in mechanisms that the 2 error sources stimulate.

A final point must be made that the current study only examines the between-trial influence of TPEs. That is, we only probed how errors experienced on the current trial influence hand direction on the next trial. In contrast, studies that have focused on within-trial, online corrections to TPEs induced through target shifts indicate that such corrections may be automatic and occur even without conscious awareness [35–38]. In other words, online corrections to jump-induced TPEs may well be implicit. However, one of the main points of the current work is that between-trial learning induced by TPEs shows hallmarks of explicit, deliberative learning. This is not to say that TPE-driven learning cannot become implicit with extensive practice; this is indeed a feature of other forms of motor learning [39,40].

The distinction between learning processes that limb-related SPEs and goal-related TPEs stimulate suggests that these mechanisms are likely neurally separable as well. While prediction-error-based implicit learning depends on the cerebellum [2,3] and parietal cortex [1,41,42], performance errors activate reward-sensitive cortico-striatal pathways [26,43]. Failure to obtain reward could trigger re-aiming via processes dependent on M1 and premotor cortex [44,45]. Changes in these regions following learning [46,47] may thus partially reflect changes in action plans driven by such processes. Future work could probe the communication between these 2 systems, which, neuroanatomically, could be sustained by reciprocal connections between the basal ganglia and the cerebellum [48].

## Materials and methods

### Participants

A total of 115 healthy, right-handed adults (85 males, 30 females, age range: 18 to 40) participated in the study. None of the participants reported any neurological, orthopedic, or cognitive impairments. All subjects gave written informed consent and were monetarily compensated for their time. The project was approved by the Institute Ethics Committee of the Indian Institute of Technology Gandhinagar and study procedures followed the principles expressed in the Declaration of Helsinki.

### Apparatus

The experimental setup consisted of a virtual reality system wherein participants sat facing a digitizing tablet (GTCO Calcomp, Scottsdale, AZ) and used a stylus to make hand movements on it (Fig A in S1 Text). A high-definition display was mounted horizontally above the tablet and was used to show circular start positions and targets for the reach, as well as a feedback cursor that would typically indicate the hand (stylus) location on the tablet. Participants looked into a mirror which was placed between the display and the tablet, and which reflected the display screen. The mirror also functioned to block direct vision of the arm. This arrangement enabled us to dissociate motion of the feedback cursor from that of the hand. For instance, cursor feedback could be veridical with the hand, “clamped” in certain directions independent of hand movement direction, or eliminated altogether.

### Task procedure and experimental design

The general task involved making center-out reaching movements from a fixed start circle to a target. To initiate a trial, participants first moved their hand (cursor) into the start circle. After 500 ms, the reach target was displayed along with a beep, which indicated to subjects that they should begin moving. No constraint was placed on reaction time or movement time. Targets were presented at a distance of 10 cm and could appear at one of 4 locations arranged radially around a virtual circle in 90-degree increments (0°, 90°, 180°, 270°). The order of target appearance was pseudo-random; a target appeared in one of the 4 locations only once over 4 consecutive trials. This order was maintained across all participants. Cursor feedback, whenever provided, was shown continuously (throughout the movement) for a distance of 10 cm at which point the cursor “froze” and stayed in place even though the hand could continue moving.

#### Experiments 1 and 2

In Experiments 1 and 2, subjects performed 3 continuous blocks of trials: baseline, learning, and washout. The baseline and washout blocks comprised of 2 sub-blocks each. In the first “no-feedback” baseline sub-block (20 trials), the cursor was not shown during the reach, while in the second “feedback” sub-block (20 trials), the cursor was shown veridical with the hand. Following baseline, subjects experienced 240 learning trials, which were followed by the 2 washout sub-blocks that were identical to baseline (20 no-feedback, 20 veridical feedback trials). Each subject thus performed 320 trials in all. During the baseline and washout blocks, the originally displayed target (at one of the 0°, 90°, 180°, 270° locations) remained “stationary,” i.e., its location did not change during the trial. During the learning block however, the target was displaced, or “jumped,” by 10° in the counterclockwise direction on every trial. This was achieved by extinguishing the originally presented target as the hand reached mid-way to it, and displaying a new target at the 10°, 100°, 190°, or 280° locations. Importantly, on these trials, the motion of the feedback cursor was always “clamped” in the direction of the original target. In other words, the cursor always followed a direct, straight path to the location of the original target regardless of the direction of hand motion.

#### Experiment 1

In Experiment 1, a start circle of 0.9 cm diameter and targets of 0.98 cm diameter were used. Participants were randomly divided into 2 groups, “Miss” or “Hit” (*n* = 15 each), which differed in terms of the learning trials experienced. For the Miss group, the original and the new target were of the same size, but for the Hit group, the diameter of the new target was increased from the original 0.98 cm to 4.6 cm (Fig B in S1 Text). Since cursor motion was always clamped towards the original target, the target jump resulted in the cursor missing the new target for the Miss group, thereby resulting in a task performance failure (TPE). However, for the Hit participants, the increase in target size ensured that the cursor would still hit the new target, thus preventing the task error. This hit was obviously not in the center of the new target.

Subjects were explained the task conditions prior to start of the experiment. They were instructed to reach towards the target on the screen. For baseline and washout trials, this obviously meant the original, fixed target. However, for learning trials, since the original target was extinguished and a new one displayed in an easily predictable location, we expected subjects to start aiming in the direction of the new target. However, no specific attempt was made to either dissuade or encourage this behavior. In addition, subjects were also informed and made to understand that on the jump (learning) trials, cursor motion would be fixed and would not depend on the direction of their hand movements. They were also explicitly told to ignore this cursor feedback. A reminder to this effect was provided at the halfway point of the learning block.

#### Experiment 2

The overall task design for Experiment 2 remained identical to Experiment 1. We recruited 2 groups of subjects (*n* = 15 each), who experienced target jumps of different amplitudes (10° or 20°) while the cursor remained clamped towards the original target. This led to conditions similar to the Miss group of Experiment 1 where the cursor did not strike the new, shifted target thus creating TPEs. Critically however, both groups of subjects were now also explicitly instructed to ignore the change in target location and reach towards the original target location. As in Experiment 1, subjects were reminded of this halfway into the learning block.

#### Experiment 3

In Experiment 3, subjects performed 400 center-out reaching movements from a start circle (1.2 cm diameter) to 4 different targets (1.5 cm diameter) in 3 continuous blocks: baseline (40 trials with veridical cursor feedback), learning (200 trials), and washout (40 no-feedback and 120 veridical feedback trials). In the learning block, the cursor was visible, but was rotated and clamped at 30° counterclockwise relative the target direction. That is, the cursor always followed a fixed, rotated path relative to the target independent of the direction of the underlying hand motion.

Participants of Experiment 3 were randomly divided into 2 groups, “Clamp” and “Clamp+Jump” (*n* = 15 each) that differed in terms of the learning trials experienced. While the 30° cursor clamp was enforced for both groups during learning, the reach target was also jumped for the “Clamp+Jump” participants to a location that was 30° counterclockwise to the original target location. This was not the case for the “Clamp” group, for whom the original target remained “stationary” and visible on the screen throughout. Given this arrangement, the Clamp subjects always experienced both an SPE and a TPE since the rotated, clamped cursor never hit the target. In contrast, the Clamp+Jump participants experienced an SPE due to the clamp, but no TPE since the cursor always landed on the shifted target at the end of the stipulated movement distance. The Clamp subjects were instructed to ignore the cursor and reach to the on-screen target, while the “Clamp+Jump” subjects were additionally asked to ignore the shift in target location and continue reaching to the original target location.

#### Experiment 4

In our fourth experiment, subjects once again performed 3 blocks of centre-out reaching trials: baseline, learning, and washout. Subjects were randomly divided in 2 groups. One of the groups (*n* = 10) performed 40 baseline trials (20 no-feedback, 20 feedback), 240 learning trials, and 40 washout trials (20 feedback, 20 no-feedback) from a start circle (0.9 cm diameter) to 4 different targets (0.98 cm diameter). The second group (*n* = 15) performed 40 feedback baseline trials, 200 learning trials, and 160 washout trials (40 no-feedback, 120 feedback) from a start circle of 1.2 cm diameter to targets of 1.5 cm diameter. For both groups, cursor feedback, when shown, was always veridical with the hand. However, subjects in the first group experienced a 10° counterclockwise target jump on the learning trials, while subjects in the second groups experienced a 30° clockwise target jump. Given that the cursor was always veridical with the hand, no SPE occurred. However, the target shift would induce a consistent TPE during the learning block in both groups.

Participants in both groups were instructed to reach to the target on the screen. This meant the originally displayed target on the baseline and washout trials, and the new, shifted target on the learning trials. Importantly, at the end of the learning block and prior to start of the washout block, participants in the 30° jump group were given the additional instruction that the target would stop jumping and that they should reach to the original target location. Such an instruction was not given to the 10° jump group.

### Mathematical modeling

We used different variants of the state-space model framework of motor adaptation to better understand our behavioral findings, particularly those of Experiment 1, and make predictions about subsequent experiments [16]. The model equations are typically of the following form:

X(n+1)=A*X(n)+B*e(n),

where *e* represents the error on the n^th^ trial, X represents the motor output, and A and B represent a retention factor and error sensitivity, respectively.

#### Independent error model

First, we considered a framework in which both SPEs and TPEs drive independent implicit processes. The net motor output reflects the sum of these SPE- and TPE-based processes. The model equations are the following:

$$X(n)=X_{SPE}(n)+X_{TPE}(n)$$

$$X_{SPE}(n+1)=A_{SPE}*X_{SPE}(n)+B_{SPE}*SPE$$

$$X_{TPE}(n+1)=A_{TPE}*X_{TPE}(n)+B_{TPE}*TPE.$$

In Experiment 1, in the Hit condition, the TPE-based update does not occur (since TPE = 0) and learning can be presumed to be driven only by the SPE-based process. However, for the Miss case, both processes get updated since the SPE and TPE are both present, and the net output is the sum of these 2 processes.

We first fit only the SPE-sensitive process of the Independent Error model to the cycle-wise data of the Hit group using the *fmincon* function of Matlab. Additionally, B_SPE_*SPE was estimated as a single parameter since the SPE remained fixed due to the clamp. A_SPE_ was the other parameter estimated from the fit. Data from the baseline, learning and no-feedback washout blocks were included, with the B_SPE_*SPE term being set to zero for the no-feedback washout cycles. The feedback washout data were not used since the SPE was no longer constant in this condition (clamp was removed) and could change as subjects changed their hand direction. The estimated values of A_SPE_ and B_SPE_*SPE derived from model fits to the Hit participants’ data were then used while fitting the model that included the TPE-based process to the data of the Miss participants of Experiment 1. Thus, only A_TPE_ and B_TPE_*TPE terms were estimated from these latter fits. For both fits, A_SPE_ and A_TPE_ were constrained between 0 and 1, while B_SPE_*SPE and B_TPE_*TPE were constrained between 0 and 10°. The 10° value corresponds to the maximum “error” (angle between new target direction and clamped cursor direction) on any given trial.

#### Interaction model

In our second “Interaction” model, TPEs cannot by themselves induce implicit learning, but can only modulate implicit learning induced by SPEs. The equations governing the trial-by-trial updates to motor output can be given by the following:

$$X(n+1)=G_{A}*A_{SPE}*X(n)+G_{B}*B_{SPE}*SPE(n).$$

Here, X represents the internal state, SPE signifies the sensory prediction error, A and B are the retention factors and error sensitivity respectively, and G_A_ and G_B_ represent parameters that modulate them. When fitting to the data from Experiment 1, for the Miss group, G_A_ and G_B_ can be set as 1, while they can be estimated from model fits for the Hit group (alternatively, G_A_ and G_B_ can also be set as 1 for the Hit group and estimated for the Miss group). Further, since the SPE remains constant in the error clamp, B*SPE is estimated as a single term.

For Experiment 1, we first fit the model to the data of the Miss group and estimated the values of A_SPE_ and B_SPE_*SPE (G_A_ and G_B_ were set to 1). We then used these estimates when fitting the model to the data of the Hit group and estimated the values of G_A_ and G_B_. The same procedure was followed in Experiment 3. The model was first fit to the data of the Clamp group (with G_A_ and G_B_ set to 1) and the values of A_SPE_ and B_SPE_*SPE were estimated. These values were then used when fitting the model to the Clamp+Jump group to estimate G_A_ and G_B_.

### Empirical data analysis and statistics

Hand position data (X-Y coordinates) were filtered using a low-pass Butterworth filter with 10-Hz cutoff frequency. Velocity values were obtained by differentiating the position data. The primary dependent variable was the deviation in hand direction relative to the original target direction. This was computed as the angle between the line connecting the start position to the original target, and the line connecting the start position to the hand position at peak movement velocity. We also calculated RT as the time elapsed between presentation of the go “beep” and movement initiation. Movement initiation was defined as the point at which hand velocity first crossed 5% of the peak velocity during the trial. Trials in which participants did not initiate a movement or lifted the stylus off the tablet mid-trial leading to loss of data were marked as “bad trials” and excluded from the analysis. Additionally, trials in which hand deviation was more than 85° were also removed. Collectively, across the 115 participants, 1.41% trials were excluded. We then calculated baseline directional biases, defined as the mean hand deviation across all baseline trials. These biases were subtracted from the trial-wise hand deviation data.

Learning was quantified using the cycle-by-cycle values of the baseline subtracted hand deviation over the learning block (1 cycle = 10 trials). For each subject, early learning was defined as the mean deviation over the first 10 learning trials while late learning was characterized by the mean deviation over the last 10 learning trials. RT during early learning was generally assessed in terms of a change from late baseline levels. This was done by subtracting the mean RT of the last 10 baseline trials from the mean RT of the first 10 learning trials. Early after-effect magnitude was quantified as the mean deviation over the first 10 trials of the no-feedback washout block. To assess whether they were sustained, average after-effect magnitude on the first 10 trials of the feedback washout block was also calculated.

Group differences in hand deviation and reaction time during early and late learning as well as the different after-effect stages were compared using Welch’s *t* tests if the normality assumption, assessed with the Shapiro–Wilk test, was violated. Paired *t* tests were used for assessing changes in hand deviation across different time points within a group. Significance levels were set at 0.05. Cohen’s *d* was used for estimating the effect size of the differences. We also ocassionally used the 95% confidence interval to probe for significant deviation in hand angle during early and late learning as well as the different after-effect stages. Lack of significant differences were augmented with Bayes factors.

## Supporting information

S1 Text Nature of the experimental setup and trials. (DOCX)

S2 Text Relationship between early and late changes in hand angle. (DOCX)

S3 Text Direct comparison of after-effects in Experiments 1 and 4. (DOCX)
