# Supplementary material for: Limb-related sensory prediction errors and task-related performance errors facilitate human sensorimotor learning through separate mechanisms
Source: PLoS Biol. 2024 Jul 3;22(7):e3002703. doi: 10.1371/journal.pbio.3002703 (PMC11221701; doi:10.1371/journal.pbio.3002703)
Supplement: S1 Text — (DOCX) [file pbio.3002703.s001.docx]

S1 Text

Nature of the experimental setup and trials


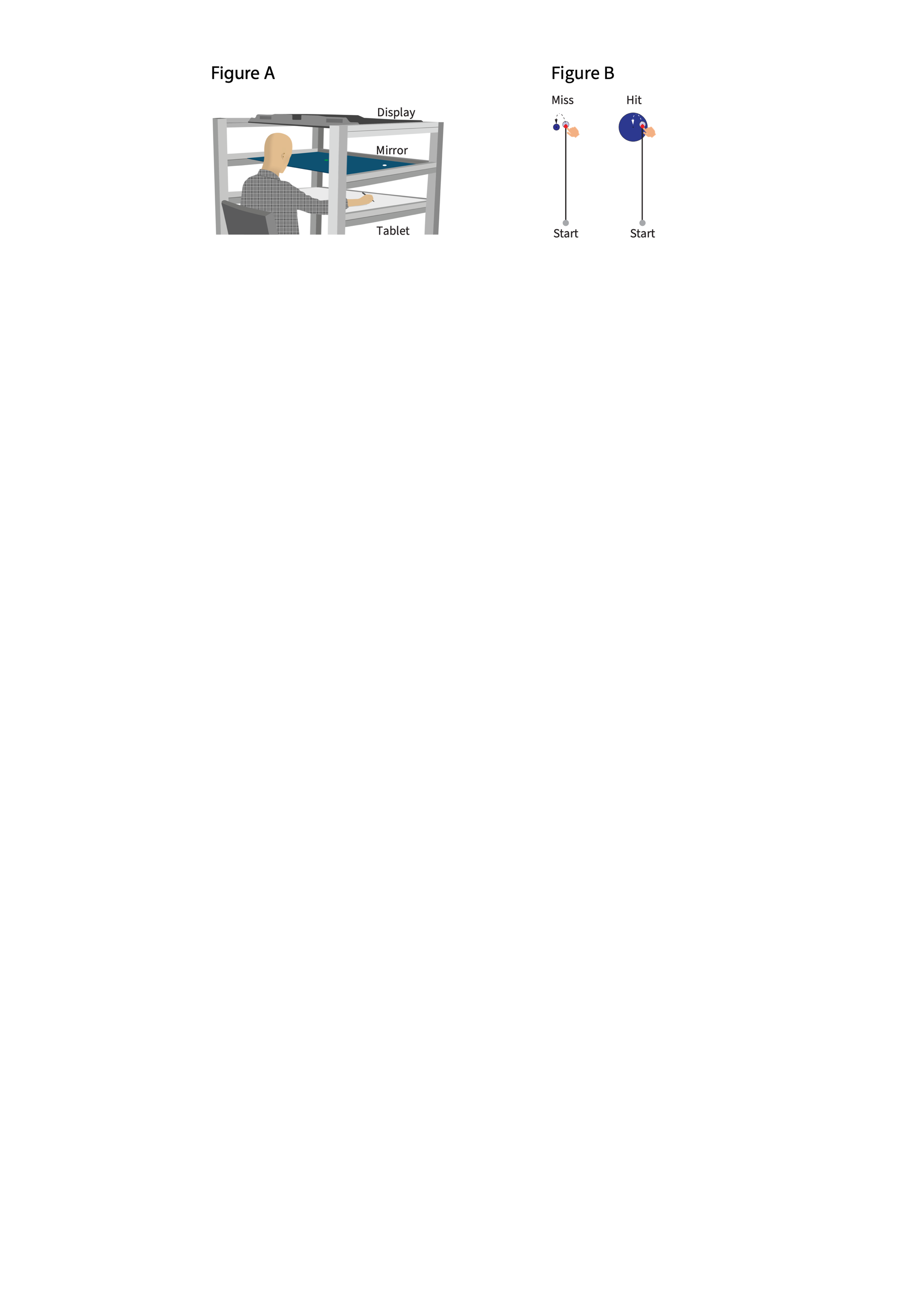


*Fig A: Subjects performed reaching movements on a digitizing tablet using a handheld stylus while looking into a mirror placed between the tablet and a horizontally mounted display screen. Movements were restricted to the horizontal plane. Start positions, targets, and a feedback cursor were displayed on the screen, and were reflected in the mirror.*

Fig B:*On learning trials, the target was displaced, or “jumped” 10° counter-clockwise, while cursor motion was clamped in the direction of the original target. For the Miss group, this resulted in a performance failure since the cursor failed to strike the new target. For the Hit group, target size was increased along with the jump, which caused the clamped cursor to always hit the new target despite the shift in target location.*
