# Supplementary material for: Limb-related sensory prediction errors and task-related performance errors facilitate human sensorimotor learning through separate mechanisms
Source: PLoS Biol. 2024 Jul 3;22(7):e3002703. doi: 10.1371/journal.pbio.3002703 (PMC11221701; doi:10.1371/journal.pbio.3002703)
Supplement: S2 Text — (DOCX) [file pbio.3002703.s002.docx]

**S2 Text**

**Relationship between early and late changes in hand angle.**

The Miss group of experiment 1 experienced performance failures. During the early learning stage, these subjects demonstrate larger RTs and increased hand deviation (relative to baseline). As our experiment 4 and other work (1, 2) shows, such changes reflect the deployment of strategic processes that cause the hand to aim away from the original target. Importantly however, this early strategy use has no bearing on how their hand angle changes over the remainder of the learning block.


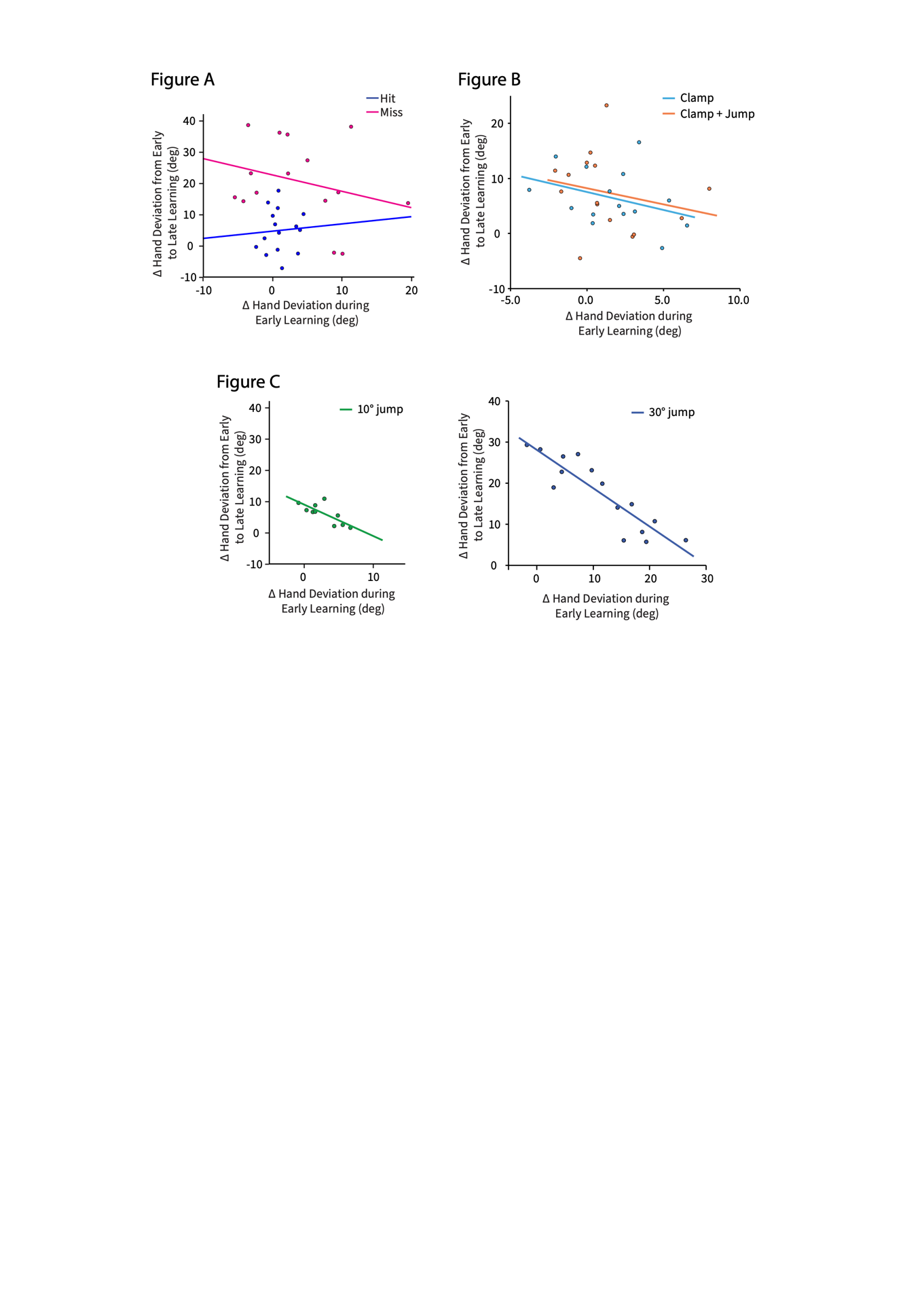


***Fig A:*** *Relationship between the change in hand deviation from baseline to early learning, and the change in hand deviation over the rest of the learning block for hit and miss groups of experiment 1.*

**Fig B:** *Relationship between the change in hand deviation from baseline to early learning, and the change in hand deviation over the rest of the learning block* for *the Clamp and Clamp+Jump groups of experiment 3.*

**Fig C:** *Relationship between the change in hand deviation from baseline to early learning, and the change in hand deviation over the rest of the learning block* for *the 10° (left) and 30° jump (right) groups of experiment 4.*

As Figure A (pink) shows, there is no relationship between the change in hand angle during early learning and the change thereafter until the end of learning. Our data suggest that the latter changes are driven by unconscious, implicit mechanisms that are engaged as subjects intend to aim to the new target location while cursor motion is clamped in the direction of the original target. Put together, our data for the Miss group suggest that the intent to aim to the new target emerges via explicit re-aiming with implicit mechanisms taking over thereafter (see elaboration in the Discussion section of the main manuscript).

The relationship between the early and later changes in hand deviation is also not seen in the Hit group (Fig A, blue). While on the face of it, this seems similar to the Miss group, the underlying reason is likely to be different. The Hit participants do not experience performance failures, and do not show any substantial change in hand angle or RT during the early learning stage. Thus, unlike the Miss group, these participants do not employ deliberative mechanisms to change their aim towards the new target early on. The changes that emerge over the course of the learning block then are likely driven entirely by an implicit process that is engaged as they gradually start aiming to the center of the new target while the cursor remains clamped in the original target direction. Thus, the absence of a relationship between early and late changes in the Hit group is because a single process that emerges slowly is engaged.

Likewise, the subjects of experiment 3 demonstrated no relationship between early deviation in hand angle and changes over the rest of the learning block (Fig B). The reason here is plausibly similar to that of the Hit participants of experiment 1. It is likely that subjects in both the Clamp and the Clamp+Jump groups employ a single, slower, implicit process to learn, as has been suggested in other reports that have engaged similar groups of subjects as well (3). Since early changes in hand angle are very small, and pretty much all learning occurs later, no relationship emerges between the early and subsequent changes in hand deviation.

In sharp contrast, a strong negative relationship between the early change in hand angle and subsequent changes is seen for the groups in Experiment 4 (Fig C). Note that these groups do not experience an SPE, and changes in hand angle are driven only by a TPE induced by the target jump. The observed relationship therefore indicates that if the early process “siphons off” a large chunk of the error, little additional change in hand angle occurs. We suggest, based on the consistent increase in RT and hand angle during early learning as well as the labile after-effects, that this process is a conscious, re-aiming strategy. Since the error is compensated via this mechanism and a prediction error is not present, an additional (implicit) learning mechanism is not engaged.

**Supporting References:**

1. J. Fernandez-Ruiz, W. Wong, I. T. Armstrong, J. R. Flanagan, Relation between reaction time and reach errors during visuomotor adaptation. *Behavioural Brain Research* **219**, 8–14 (2011).
2. S. D. McDougle, J. A. Taylor, Dissociable cognitive strategies for sensorimotor learning. *Nat Commun* **10**, 40 (2019).
3. L. A. Leow, W. Marinovic, A. de Rugy, T. J. Carroll, Task errors drive memories that improve sensorimotor adaptation. *Journal of Neuroscience* **40**, 3075–3088 (2020).
