# Supplementary material for: Limb-related sensory prediction errors and task-related performance errors facilitate human sensorimotor learning through separate mechanisms
Source: PLoS Biol. 2024 Jul 3;22(7):e3002703. doi: 10.1371/journal.pbio.3002703 (PMC11221701; doi:10.1371/journal.pbio.3002703)
Supplement: S3 Text — (DOCX) [file pbio.3002703.s003.docx]

**S3 Text**

**Direct comparison of after-effects in experiments 1 and 4.**

The Hit and Miss groups of experiment 1 showed sustained post-learning after-effects. Subjects of experiment 4, who experienced only TPEs, showed deviations in hand angle immediately after the learning block had ended, but this effect was not sustained particularly for the group that experienced the 30° jump. However, a direct comparison between these groups is precluded since the asymptotic level of learning is different between them. To overcome this issue, we used the following approach. We considered only the hand angle data of the last learning cycle and the four washout cycles (two no-feedback, two feedback washout cycles). We then scaled the data of individual Hit and Miss participants of experiment 1, and the subjects who experienced the 10° jump in experiment 4 to match the mean hand angle of the 30° jump group on the last learning cycle. Thus, at the end of this step, the mean hand angle at the end of learning for all four groups was identical. We then compared the (scaled) after-effects across the four groups (Fig A).


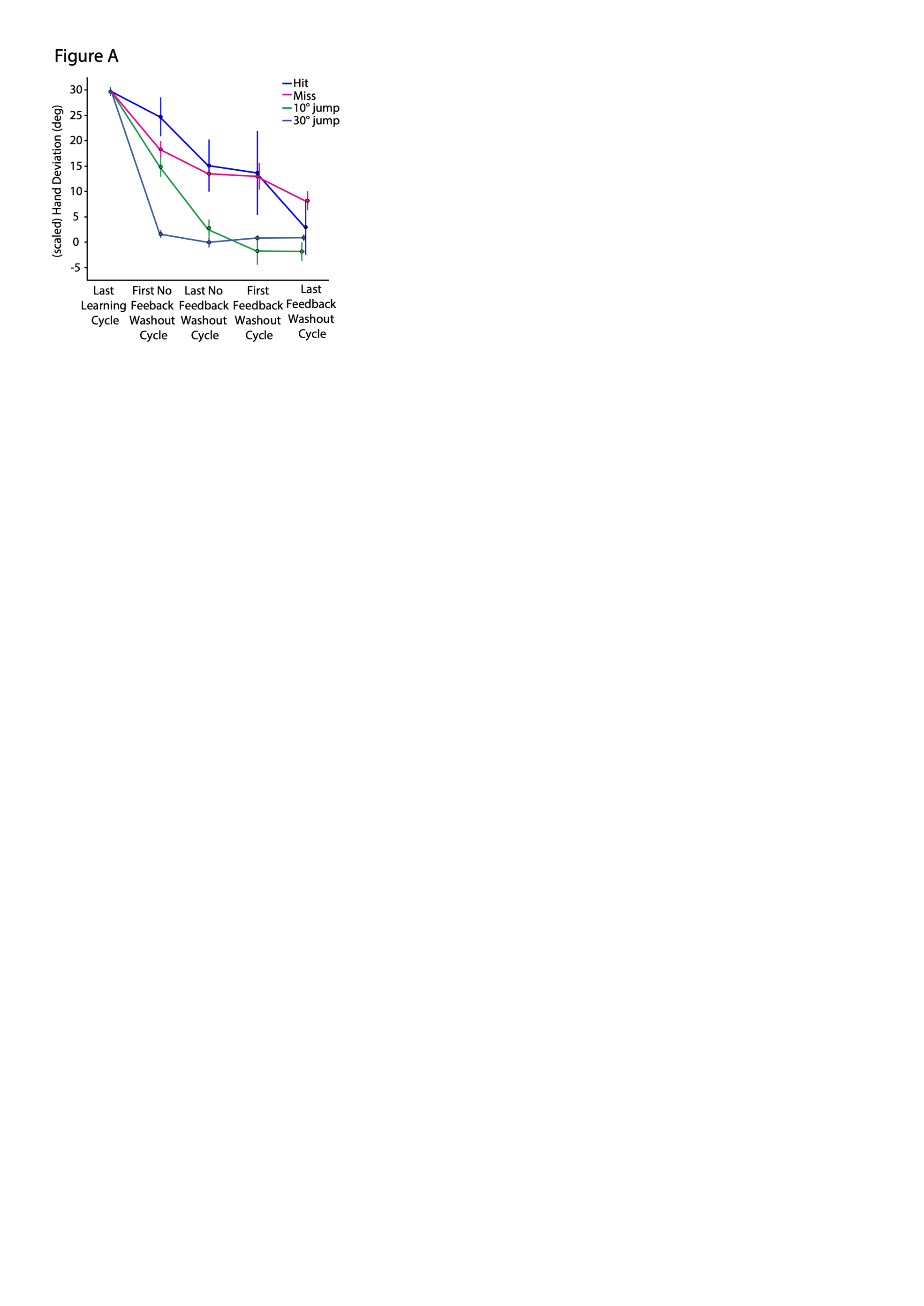


***Fig A:*** ***Direct comparison of after-effects in experiment 1 and experiment 4.*** *Scaled average hand deviation of the Hit and Miss groups of experiment 1 and the 10*° *and 30*° *jump groups of experiment 4. Data on the last learning cycle, the two no-feedback washout cycles and the two feedback washout cycles are shown. Error bars represent SEM. The (scaled) hand deviation on washout trials decayed more rapidly towards zero for the subjects of experiment 4.*

We found a clear group difference in the hand deviation on the first no-feedback washout cycle (F(3,51) = 17.671, p < 0.001, η^2^_p_ = 0.510). A Dunnett’s post-hoc test showed that relative to the 30° jump group, the three other groups, i.e., 10° jump (p=0.002), Miss (p<0.0001), and Hit (p<0.0001) had much larger had deviation. Group differences were also prevalent during the later no-feedback washout trials (F(3,51) = 5.918, p = 0.001, η^2^_p_ = 0.258), but Dunnett’s test now indicated a significantly greater hand angle for only the Hit (p = 0.0027) and Miss (p = 0.0080) groups, but not the 10° jump group (p = 0.9196) relative to the 30° jump group. This suggested that subjects in the 10° jump group had also returned to near baseline levels of hand deviation by this time. The group differences began to diminish as washout trials progressed; there was no major difference between the groups on the early feedback washout trials (F(3,51) = 2.592, p = 0.063, η^2^_p_ = 0.132), and group differences were ngliglible at the end of washout (F(3,51) = 1.613 , p = 0.198, η^2^_p_ = 0.087).

As we have argued in the main manuscript, the subjects in experiment 4 (10° and 30° jump) compensated for the imposed TPE by employing a deliberative re-aiming strategy. As such, this strategy (of aiming away from the original target) would be irrelevant on washout trials. The highly transient nature of after-effects suggests that strategy use was rapidly “abandoned” by these subjects when it was no longer relevant. This was particularly the case for the 30° jump group that in fact was explicitly instructed before the washout trials that the target would stop jumping and they should move to the original target location. The 10° jump group also shows a rapid decline, with their hand deviation returning to baseline levels by the second washout cycle (this group was not given any instruction before washout). However, the Hit and Miss groups return to baseline levels much more slowly since their learning is dominated by SPE-driven implicit mechanisms.
